# Supplementary material for: Stratified Effects of Tillage and Crop Rotations on Soil Microbes in Carbon and Nitrogen Cycles at Different Soil Depths in Long-Term Corn, Soybean, and Wheat Cultivation
Source: Microorganisms. 2024 Aug 10;12(8):1635. doi: 10.3390/microorganisms12081635 (PMC11356494; doi:10.3390/microorganisms12081635)
Supplement: Supplementary file 1 [file microorganisms-12-01635-s001.zip › microorganisms-3140528-supplementary.pdf]

Article

Stratified Effects of Tillage and Crop Rotations on Soil Microbes in Carbon and Nitrogen Cycles at Different Soil Depths in Long-Term Corn, Soybean, and Wheat Cultivation

Yichao Shi[1], A. Claire Gahagan[1], Malcolm J. Morrison[1], Edward Gregorich[1], David R. Lapen[1], Wen Chen[1,2*]

[1] Ottawa Research and Development Centre, Agriculture and Agri-Food Canada, 960 Carling ave., Ottawa, ON K1A 0C6, Canada
[2] Department of Biology, University of Ottawa, Ottawa, 60 Marie Curie Prv., Ottawa, ON K1N 6N5, Canada
* Correspondence: wen.chen@agr.gc.ca

Supplementary documents

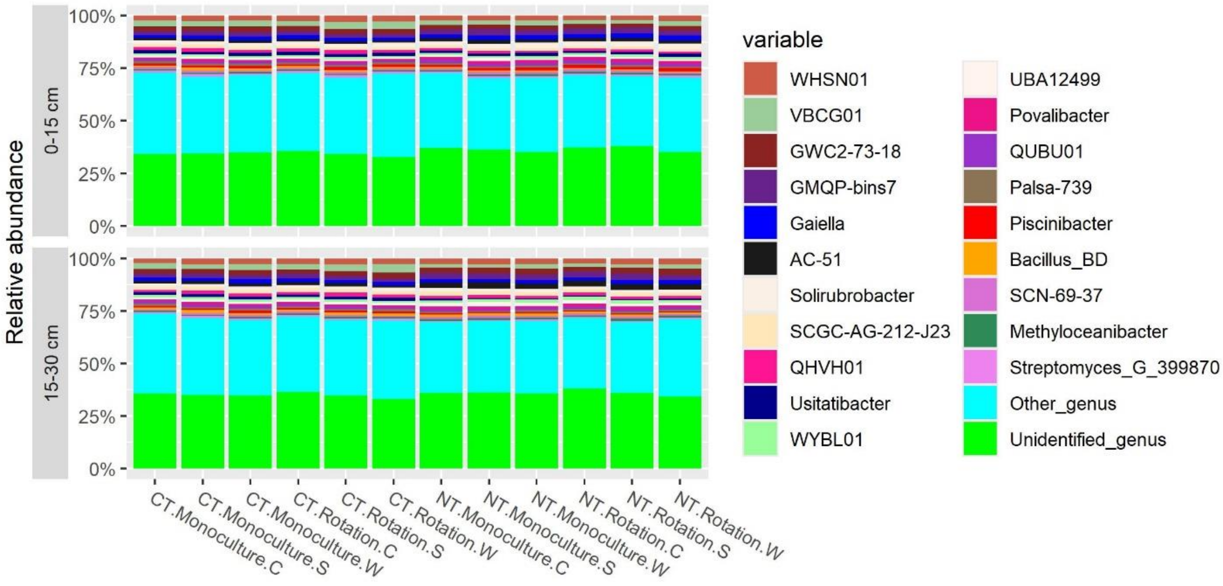

Figure S1. Relative abundance of the top 20 soil bacterial genera at two soil depths (0-15 cm, 15-30 cm). CT, conventional tillage; NT, no-till; C, corn; S, soybean; W, wheat.

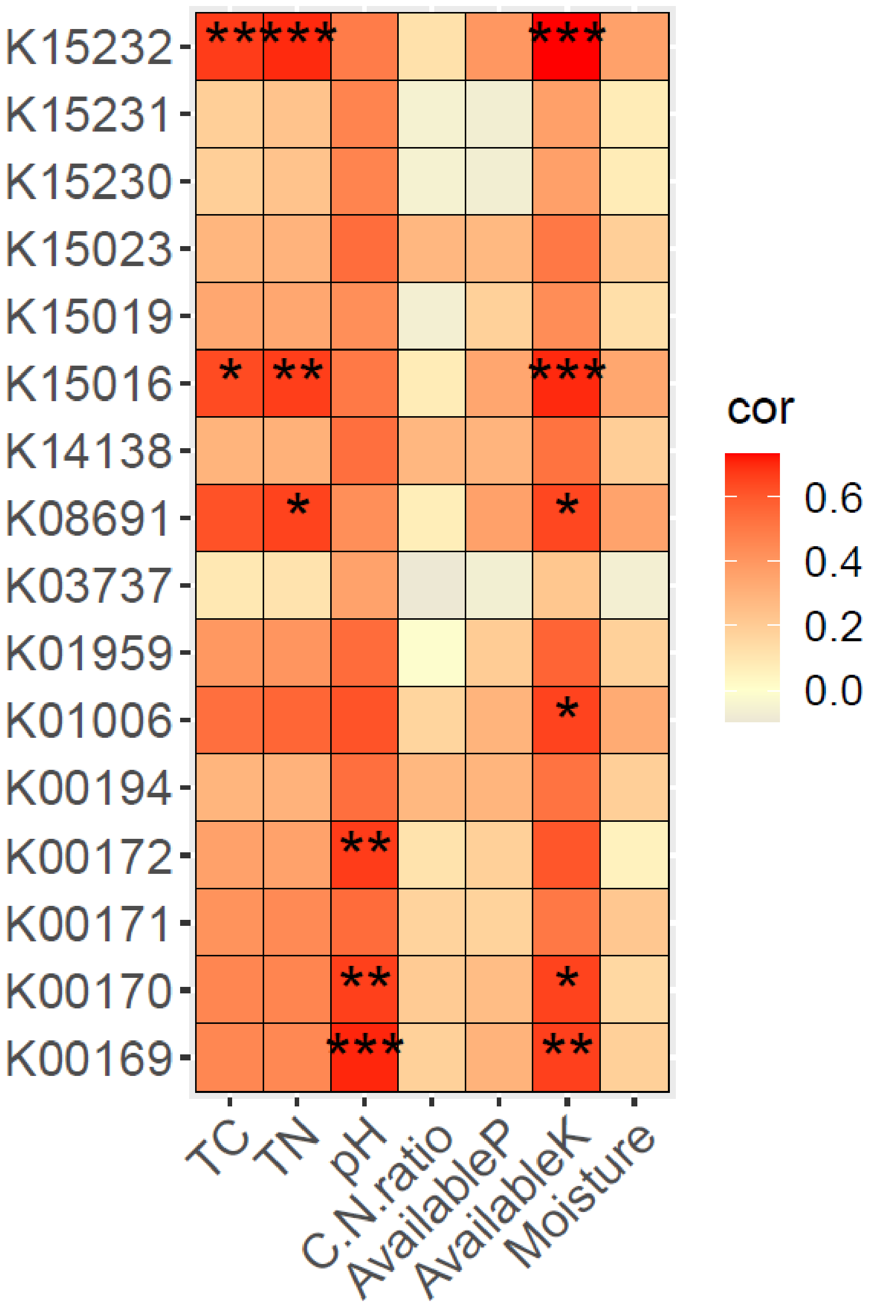

Figure S2. Correlations between KOs belonging to C fixation pathways and soil physicochemical properties at the 0-15 cm depth. The KOs were enriched by NT compared to CT. *, **, and *** represent significant differences with p = 0.05, 0.01, and 0.001, respectively.

A)

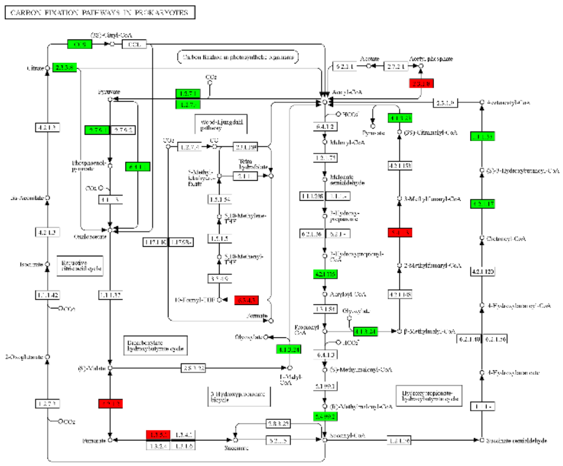
CARBON FIXATION PATHWAYS IN PROKARYOTES

B)

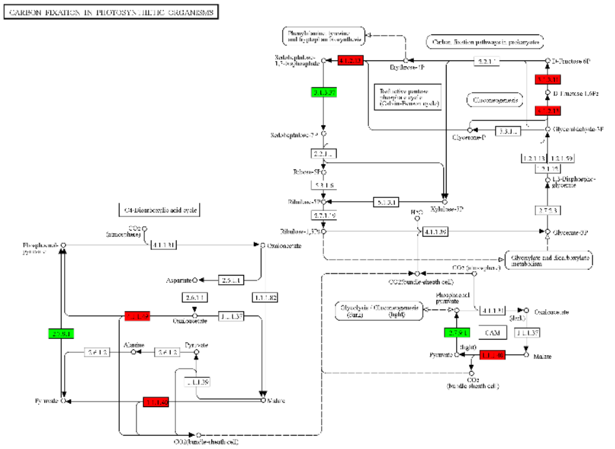
CARBON FIXATION IN PHOTOSYNTHETIC ORGANISMS

C)

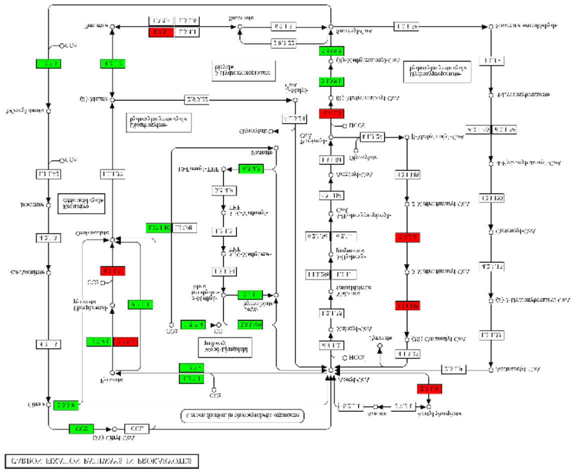

D)

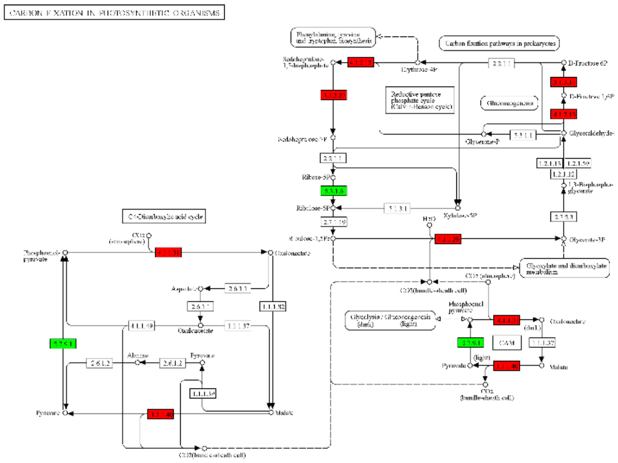
CARBON FIXATION IN PHOTOSYNTHETIC ORGANISMS

E)

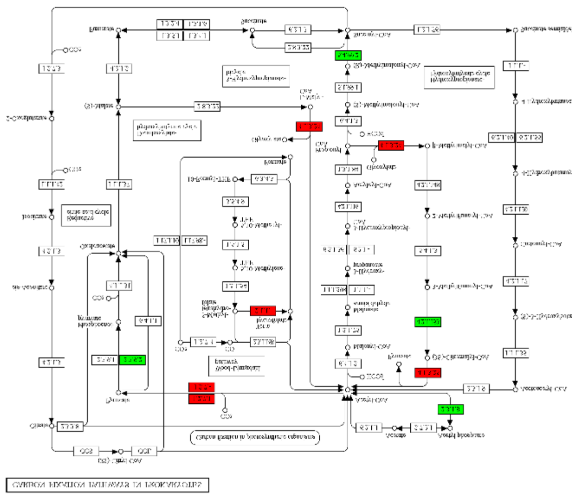

F)

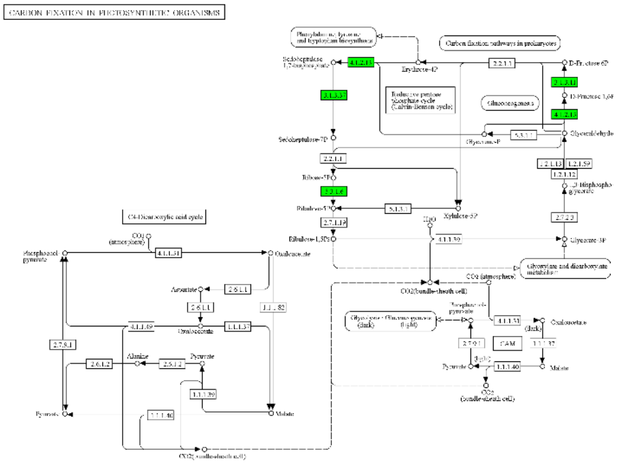
CARBON FIXATION IN PHOTOSYNTHETIC ORGANISMS

Figure S3. The KEGG pathways associated with the microbial C fixation (map00720) (A, C, E) and Calvin–Benson cycle (map00710) (B, D, F) pathways at the 0-15 cm soil depth (A, B) and the 15-30 cm soil depth (C, D), affected by tillage (A, B, C, D) and crop type (E, F). A, B, C, D). The genes with a green background were enriched by NT, while those with a red background were enriched by CT. (E, F) The genes with a green background were enriched in wheat soils, while those with a red background were enriched in corn and/or soybean soils.

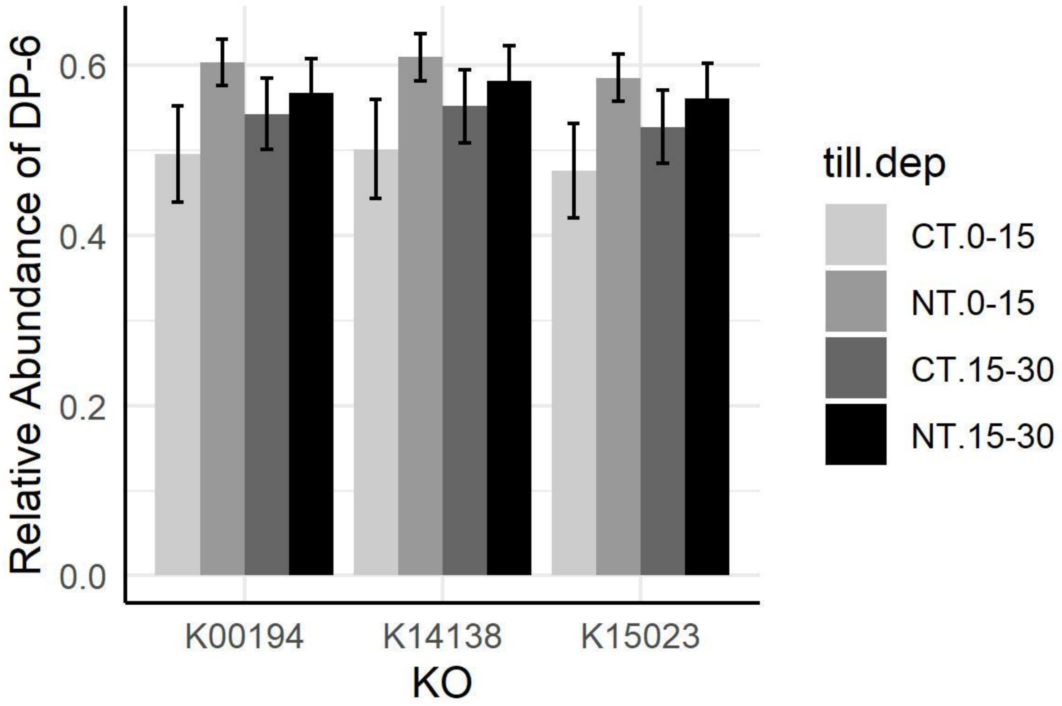

Figure S4. The relative abundance of three KOs belonging to the Wood–Ljungdahl pathway in carbon fixation identified in Candidatus DP-6, affected by tillage and soil depths. CT, conventional tillage; NT, no-till; 0-15, 0-15 cm soil depth; 15-30, 15-30 cm soil depth. Error bars represent standard errors.
